# Predicting the Impact of Vaccination on the Transmission Dynamics of Typhoid in South Asia: A Mathematical Modeling Study

**DOI:** 10.1371/journal.pntd.0002642

**Published:** 2014-01-09

**Authors:** Virginia E. Pitzer, Cayley C. Bowles, Stephen Baker, Gagandeep Kang, Veeraraghavan Balaji, Jeremy J. Farrar, Bryan T. Grenfell

**Affiliations:** 1 Department of Epidemiology of Microbial Diseases, Yale School of Public Health, New Haven, Connecticut, United States of America; 2 Fogarty International Center, National Institutes of Health, Bethesda, Maryland, United States of America; 3 Department of Epidemiology, Harvard School of Public Health, Boston, Massachusetts, United States of America; 4 Department of Ecology and Evolutionary Biology, Princeton University, Princeton, New Jersey, United States of America; 5 The Hospital for Tropical Diseases, Wellcome Trust Major Overseas Programme, Oxford University Clinical Research Unit, Ho Chi Minh City, Vietnam; 6 Centre for Tropical Medicine, Nuffield Department of Clinical Medicine, Oxford University, Oxford, United Kingdom; 7 The London School of Hygiene and Tropical Medicine, London, United Kingdom; 8 Department of Gastrointestinal Sciences, Christian Medical College, Vellore, Tamil Nadu, India; Massachusetts General Hospital, United States of America

## Abstract

**Background:**

Modeling of the transmission dynamics of typhoid allows for an evaluation of the potential direct and indirect effects of vaccination; however, relevant typhoid models rooted in data have rarely been deployed.

**Methodology/Principal Findings:**

We developed a parsimonious age-structured model describing the natural history and immunity to typhoid infection. The model was fit to data on culture-confirmed cases of typhoid fever presenting to Christian Medical College hospital in Vellore, India from 2000–2012. The model was then used to evaluate the potential impact of school-based vaccination strategies using live oral, Vi-polysaccharide, and Vi-conjugate vaccines. The model was able to reproduce the incidence and age distribution of typhoid cases in Vellore. The basic reproductive number (*R*_0_) of typhoid was estimated to be 2.8 in this setting. Vaccination was predicted to confer substantial indirect protection leading to a decrease in the incidence of typhoid in the short term, but (intuitively) typhoid incidence was predicted to rebound 5–15 years following a one-time campaign.

**Conclusions/Significance:**

We found that model predictions for the overall and indirect effects of vaccination depend strongly on the role of chronic carriers in transmission. Carrier transmissibility was tentatively estimated to be low, consistent with recent studies, but was identified as a pivotal area for future research. It is unlikely that typhoid can be eliminated from endemic settings through vaccination alone.

## Author Summary

We developed a mathematical model for the transmission dynamics of typhoid in order to evaluate the potential direct and indirect (i.e. herd immunity) effects of vaccination. The model was fit to data from Vellore, India and validated against the results of cluster randomized vaccine trials. We evaluated a variety of school-based vaccination strategies and found that typhoid vaccination is expected to lead to short-term indirect protection and a decrease in typhoid incidence, but vaccination alone is unlikely to lead to elimination. The level of indirect protection was found to depend on assumptions about the role of chronic carriers, identifying an important area for future research.

## Introduction

Typhoid has a long and storied history as a public health scourge. *Salmonella enterica* serovar Typhi (*S.* Typhi) is a human-restricted bacterial pathogen transmitted via faecal contamination of food and water. While improvements in water and sanitation led to the elimination of typhoid from most developed countries during the twentieth century, the global burden of typhoid fever has recently been estimated to be between 13.5 and 26.9 million episodes and 190,000 to 216,000 deaths annually [1]–[3].

Children in South Asia carry one of the highest typhoid burdens in the world [1], [4]. The emergence of antimicrobial resistance has further compounded the problem, with treatment failure and complications becoming more common [5]. Several public health authorities have suggested school-based vaccination programs as a means of control [6]. Such programs have been successfully carried out in Thailand, China, and Viet Nam [7], [8].

There are two widely licensed vaccines against typhoid [9]. Ty21a is a multi-dose live oral vaccine, inducing both cellular and antibody-mediated immune responses [10], while Vi polysaccharide (ViPS) is a single-dose injectable vaccine that induces an antibody response to the capsular Vi antigen [11]. Both vaccines demonstrated moderate efficacy in field trials [12], [13]. Notably, neither is licensed for use in children <2 years old. There are also several Vi conjugate vaccines (ViCV) in advanced stages of development. Existing data predicts that ViCV can be effectively administered to infants and will elicit a stronger and longer-lasting immune response than ViPS [14], [15].

Cluster randomized trials suggest that typhoid vaccination may induce some herd immunity, but results have been variable [16], [17]. Mathematical models of typhoid transmission dynamics rooted in data can help interpret the trial results and generalize the findings to the long-term impact of vaccination at various coverage levels, but few such models currently exist [18]–[21]. Mathematical modeling is highly pertinent for deciding how ViCV can be best utilized when they become widely available.

We developed a model for the transmission dynamics of typhoid to quantify the expected population-level impact of vaccination. We fit our model to a 12-year time series of culture-confirmed cases from Vellore, India to estimate important unknown parameters. We then examined the potential impact of vaccination under a variety of assumptions about vaccine efficacy and duration of protection, reflecting the characteristics of the currently available and next generation vaccines. We also explored how chronic carriers influenced the expected indirect and overall effects of vaccination.

## Methods

### Ethics statement

The study protocol was approved by the Oxford Tropical Research Ethics Committee.

### Data sources

Data on patients hospitalized with typhoid or paratyphoid between January 1, 2000 and February 29, 2012 were obtained from Christian Medical College (CMC) in Vellore, India. The CMC hospital is a 2,695-bed referral tertiary care facility in Tamil Nadu. We obtained data on patient age, primary and secondary diagnoses, discharge date, and whether they died. All patient identifiers were removed. We limited our analysis to patients with culture-confirmed typhoid (A01.0 according to the ICD10-CM), and aggregated the data by week of discharge and patient age in 5-year age groups. Approximately 45% of *S.* Typhi strains isolated between 1999 and 2002 were resistant to ampicillin, chloramphenicol, and co-trimoxazole, although the percentage of resistant isolates was decreasing over this time period (V. Balaji, unpublished data).

The catchment population for CMC hospital is not well defined, but likely includes a subset of Vellore district, surrounding populations, and patients referred from other states. We obtained demographic data on the population of Vellore district and the crude birth and death rates in Tamil Nadu from 1971 to 2015 (http://www.indiastat.com), and derived estimates of the weekly number of births (Text S1). To simulate the potential impact of vaccination, we assumed the birth and death rates remained at the most recently observed levels.

### Model description

**Figure 1:**
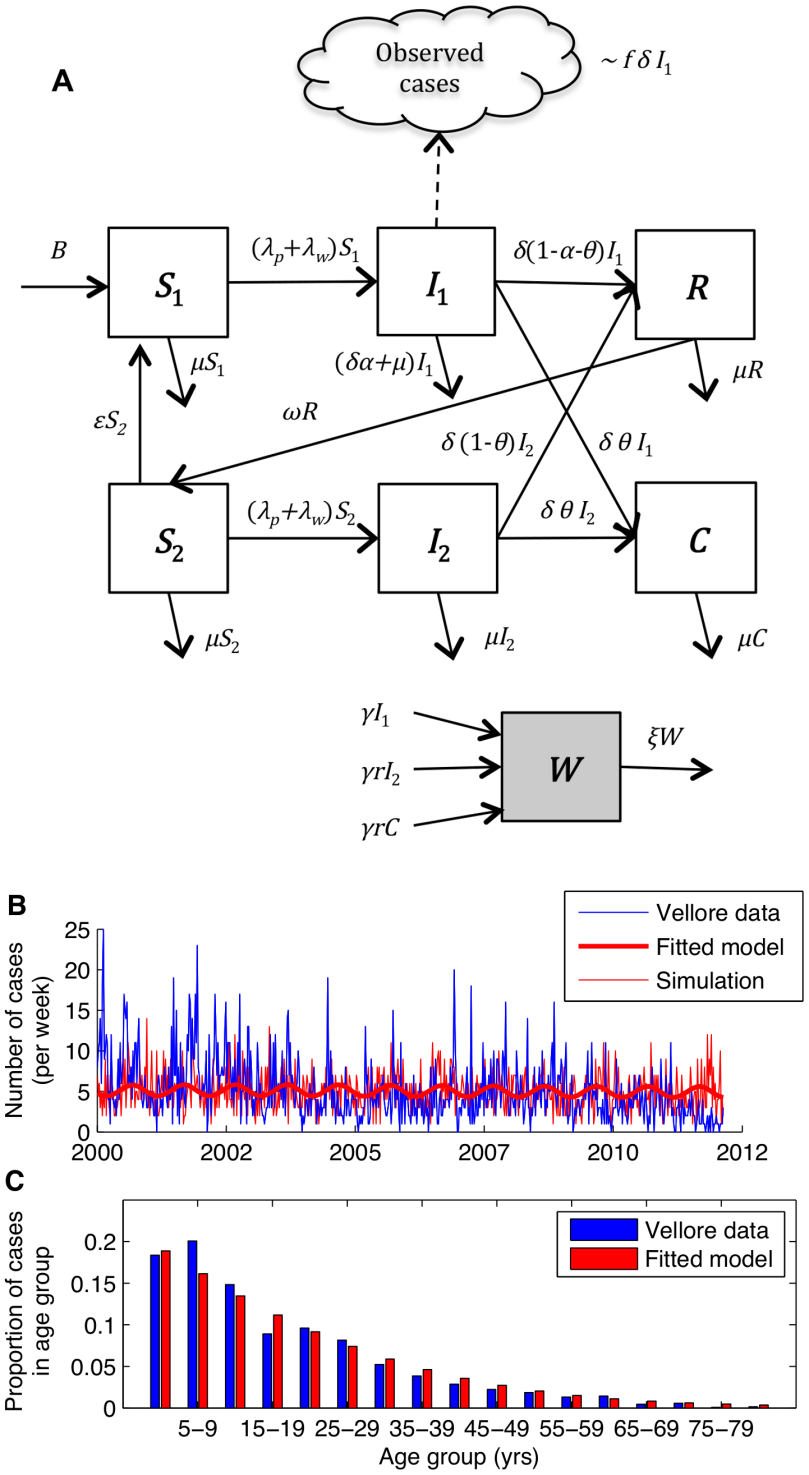
Model for the transmission dynamics of typhoid. (*A*) Diagram of model structure. Model parameters are defined in Table 1. (*B*) Weekly incidence of observed (blue line) and model-predicted (thick red line) typhoid inpatients at Christian Medical College hospital in Vellore, India. The thin red line represents a simulated incidence time-series for the best-fit model assuming the number of cases each week is Poisson distributed with a mean equal to the model-predicted incidence. (*C*) Age distribution of observed (blue) and model-predicted (red) typhoid cases.

Our model for the transmission dynamics of typhoid is illustrated in Figure 1. The essential features include: (1) a distinction between “primary infection” (*I*_1_) of fully susceptible individuals (*S*_1_), which may lead to hospitalization, and subsequent subclinical infection (i.e. short-term carriage) (*I*_2_) of partially susceptible individuals (*S*_2_); (2) loss of immunity (*R*) to subclinical typhoid infection and boosting of clinical immunity through repeated subclinical infections; (3) the inclusion of a chronic carrier state (*C*), in which a small fraction of infections lead to life-long carriage of typhoid [22], [23]; and (4) a balance between “short-cycle” transmission via contamination of food, drinking water, etc. in the immediate environment and “long-cycle” transmission via contamination of the water supply (*W*), the latter of which we assume varies seasonally. Both short- and long-term carriers contribute to transmission, but not necessarily at the same rate as primary infections.

Model parameters are described in Table 1; a full description of the model can be found in the Text S1. We fixed the value of parameters described in the literature then estimated the remaining parameters by fitting the model to the Vellore data (Table 1). The basic reproductive number (*R*_0_) can be calculated from the model parameters and broken down into its components in order to better understand the contribution of short-cycle (*R*_0,*p*_) versus long-cycle (*R*_0,*w*_) transmission, and the relative role of carriers compared to primary infections (Text S1).

**Table 1:** Fixed model parameters and parameters estimated from the best-fit model for typhoid inpatients at Christian Medical College hospital in Vellore, India.

*Fixed parameters*			
Parameter definition	Symbol	Value	Source
Birth rate	*B*	14.9–19.2 live births per 1,000	Census data
Natural mortality rate	*μ*	7.2–8.2 deaths per 1,000	Census data
Duration of infectiousness	1/*δ*	4 weeks	[36]
Fraction infected who become chronic carriers	*θ*	0.003–0.101 depending on age	[33]
Disease-induced mortality	*α*	0.001	Assumption, [1]
Duration of temporary full immunity to infection	1/*ω*	104 weeks	Assumption*, [30]
Rate of shedding into the water supply	*γ*	1 infectious unit/week	Assumption†
Rate of decay of infectious particles from water supply	*ξ*	1/3 week^−1^	[37]

*Estimated parameters*		
Parameter definition	Symbol	Estimate
Basic reproductive number for short-cycle transmission	*R_0,p_*	2.49
Basic reproductive number for long-cycle transmission	*R_0,w_*	0.28
Amplitude of seasonal forcing (long-cycle transmission)	*q*	0.69
Seasonal offset parameter (timing of seasonal peak)	*φ*	18.2 weeks
Rate of waning immunity to clinical disease	*ε*	2.68×10^−11^
Relative infectiousness of chronic and short-term carriers	*r*	0.01
Reporting fraction	*f*	0.0052

The best-fit model was not sensitive to this parameter.

Inseparable from the estimated long-cycle transmission parameter, *β_w_*.

### Model fitting and validation

We fit our model to the Vellore data by maximizing the likelihood assuming the weekly number of reported typhoid cases in each age group is Poisson-distributed with a mean equal to a fraction *f* of the model-predicted number of primary infections (Text S1). The reporting fraction *f* incorporates the probability that an individual with clinical typhoid infection in Vellore district will seek care at (or be referred to) CMC hospital, be admitted as an in-patient, and be culture-confirmed; hence, it takes into account many factors, including the probability of primary infection leading to clinical typhoid, treatment-seeking behavior, and culture sensitivity (which is relatively poor).

We validated our best-fit model by comparing predictions for the indirect and overall effectiveness of vaccination using ViPS to observations from cluster randomized trials conducted in Kolkata, India and Karachi, Pakistan [16], [17]. The indirect effectiveness is defined as the reduction in the incidence rate among unvaccinated individuals in a partially vaccinated population compared to the incidence rate in a completely unvaccinated population, while the overall effectiveness is defined as the incidence rate among vaccinated and unvaccinated individuals in a partially vaccinated population compared to the incidence rate in a completely unvaccinated population [24]. Preliminary analyses revealed that the predicted level of indirect and overall protection was strongly dependent on assumptions about the proportion of transmission due to carriers (*c_p_*). We therefore examined model predictions for vaccine effectiveness for *c_p_* from 5% to 95% by varying the relative infectiousness of carriers (*r*).

### Vaccination

We modeled vaccination in two ways to accommodate the different immune mechanisms of the live oral versus Vi-based vaccines (Figure S1, Text S1). We assumed the vaccine efficacy for Ty21a was 48% in accordance with a recent meta-analysis; waning of immunity to was assumed to mimic natural immunity [9]. For ViPS, we assumed an initial vaccine efficacy of 80% and a mean duration of protection of 3 years, while for ViCV, we assumed an initial efficacy of 95.6% and a duration of 19.2 years, based on a comparison between the predicted direct effect (i.e. reduction in the cumulative incidence among vaccinated versus unvaccinated individuals) and the waning of vaccine efficacy observed during trials (Figure S2) [9].

For all three vaccines, we examined the following school-based vaccination strategies:

- One-time campaign among school-aged children (6–15 years old)
- Routine vaccination at school entry (6 years old)
- Routine vaccination at school entry plus a one-time catch-up campaign among 6–15 year olds.

Since one of the goals of ViCV is to provide immunity from a younger age, we explored the potential impact of routine vaccination at 9 months old, with and without a catch-up campaign among 9-month to 15-year olds. We also examined the benefit of including revaccination with ViPS every three years (at 6, 9, and 12 years of age), and whether it would be possible to eliminate typhoid using ViCV and an aggressive strategy consisting of one-time mass vaccination followed by routine vaccination of infants (at 9 months old) and school-aged children (at 6 and 12 years old).

We evaluated the impact of vaccination on the projected typhoid incidence over time assuming a coverage level of 80%, and compared this to the projected incidence without vaccination and if the vaccine was assumed to provide direct protection only (i.e. the *population direct effect*, which we define as the reduction in incidence that would be predicted by a static model that did not account for the transmission dynamics of infection; Text S1). We also evaluated the overall vaccine effectiveness (i.e. fractional reduction in cumulative typhoid incidence) over the first 10 years following vaccine introduction for coverage levels of 0–100%. Finally, the expected number of typhoid cases prevented per 1,000 vaccine courses administered was calculated over 1–20 years following vaccine introduction at 80% coverage, assuming 10% of primary infections result in clinical typhoid cases.

### Comparison between the impact of vaccination and improvements in sanitation

We also explored the potential impact of improvements in water quality and sanitation, with and without vaccination, under a variety of assumptions about the proportion of transmission due to carriers (*c_p_*) and the proportion of transmission that is water-borne (and therefore potentially reduced through such broad-scale interventions). To model the impact of sanitation, *R*_0,*w*_ was reduced from its starting value (25% to 100% of *R*_0_ = 2.8) to 0 linearly over a 30-year period (years 5–35). The additional impact of vaccination was assessed for the ViPS vaccine assuming routine vaccination of 6 year olds plus a catch-up campaign among 6–15 year olds beginning in year 5 with 80% coverage.

All analyses were carried out using MATLAB version 7.14 (MathWorks, Natick, MA).

## Results

### Model fit to the observed incidence of typhoid in Vellore

The weekly incidence of culture-confirmed typhoid infections at CMC hospital varied from 0 to 25 cases per week, while the incidence of culture-confirmed paratyphoid varied from 0 to 4 cases per week (Figure S3 *A*). Thus, much of the burden of enteric fever in this population is due to *S.* Typhi and therefore could potentially be prevented by a Vi-based vaccine. Cases occurred throughout the year; there was only weak seasonal variability in incidence, which did not appear to be correlated with rainfall (Figure S3 *B*). The mean age of typhoid patients was 17.6 years, with burden generally decreasing with age (Figure S3 *C*).

Our best-fit model was able to reproduce the incidence pattern and age distribution of typhoid cases in Vellore (Figure 1*B,C*). Point estimates of key parameters were well located and remarkably insensitive to the fixed parameter assumptions, indicating that the model is not over-specified relative to the age-structured time series data used for fitting (Figure S4, Table S1). The basic reproductive number for the best-fit model was *R*_0_ = 2.8 (95% CI: 2.6, 2.9). Short-cycle transmission accounted for 90% of the *R*_0_ value (Table 1), but estimates of *R*_0,*w*_ and *R*_0,*p*_ were highly correlated (Figure S4). Following recovery from infection, most individuals were estimated to remain immune to clinical disease (*ε* ≈0). The proportion of symptomatic typhoid infections in Vellore district that seek care at CMC hospital, are admitted as inpatients and microbiologically confirmed was estimated to be low (*f* = 0.0052). Assuming 10% of primary infections result in clinical typhoid, the model-predicted an annual incidence rate of 146 cases per 100,000 person-years, which is consistent with the estimated incidence rate in Kolkata, India [16]. The prevalence of chronic carriers was predicted to increase with age (Figure S5), and was estimated to be ∼2% among 30–40 year olds, which is similar to the prevalence of carriage among patients undergoing cholecystectomy in Kathmandu, Nepal [25]. We also examined the impact of vaccination when the proportion of infections leading to chronic carriage was 90% lower, resulting in a comparable reduction in the prevalence of chronic carriers (Figure S5).

**Figure 2:**
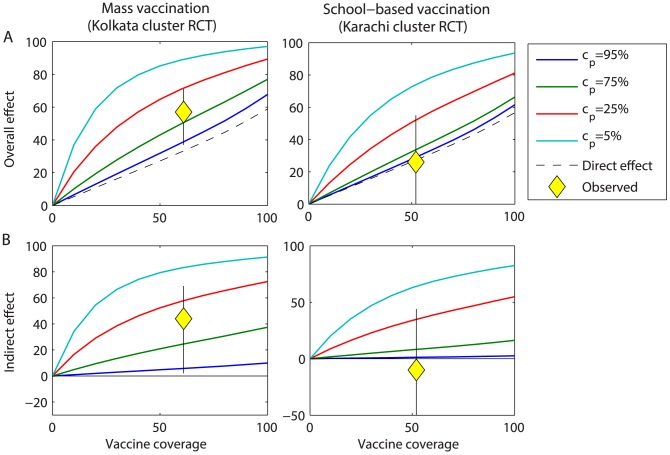
Relationship between the relative infectiousness of chronic carriers and the model-predicted overall and indirect effects of vaccination. (*A*) Overall effect and (*B*) indirect effect of ViPS over two years following vaccine introduction at coverage levels varying from 0 to 100%. The model-predicted vaccine effects over 2 years of follow-up for *c_p_* = 5 to 95% are represented by the thick coloured lines, while the population direct effect is represented by the dotted black line. Mass vaccination consisted of a one-time campaign among all individuals ≥2 years of age, while school-based vaccination consisted of a one-time campaign among 2–15 year olds. The overall and indirect effects observed during cluster randomized trials conducted in Kolkata, India and Karachi, Pakistan are plotted in yellow, with the thin black line corresponding to the 95% confidence interval [16], [17].

The model predicted that vaccination should provide little or no indirect protection if carriers were primarily responsible for driving transmission (*c_p_* = 95%) (Figure 2). While this was consistent with the levels of overall and indirect protection observed during the cluster randomized trial conducted in Karachi [17], it was not consistent with the results from Kolkata [16]. As *r* decreased, the predicted level of indirect and overall protection increased substantially. The overall effectiveness observed in Kolkata was most consistent with values of *c_p_* between 25% and 75% (corresponding roughly to *r* between 0.01 and 0.1). Therefore, *r* was constrained to be between 0.01 and 1 when fitting the model to the Vellore data. The best-fit model consistently estimated *r* to fall at the lower end of this range (*r* = 0.01), suggesting that chronic carriers play a limited role in transmission in this setting.

### Overall effectiveness and impact of vaccination

**Figure 3:**
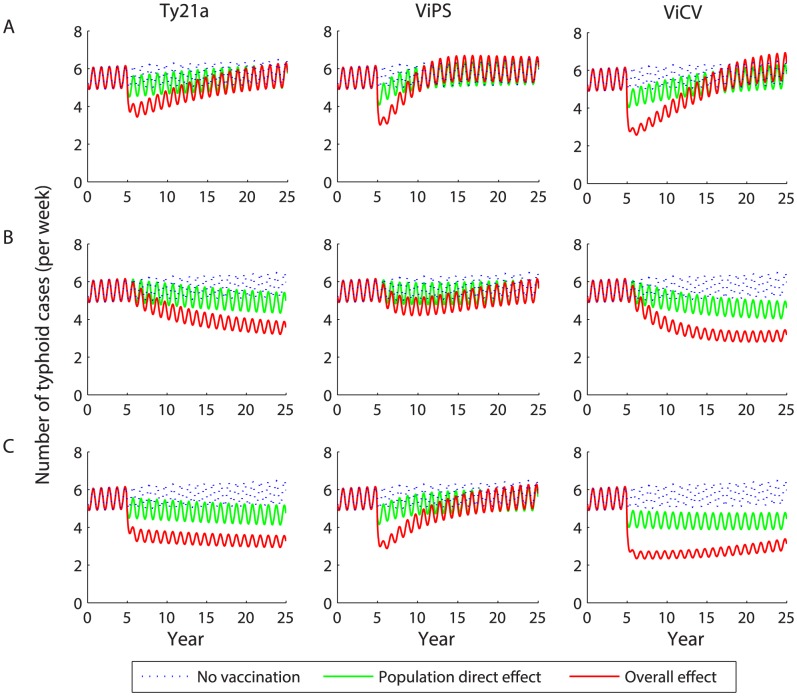
Predicted impact of vaccination on the weekly incidence of typhoid. Vaccination is introduced in year 5 with 80% coverage as (*A*) a one-time campaign among 6–15 year olds, (*B*) routine vaccination of 6 year olds, or (*C*) routine vaccination of 6 year olds plus a one-time catch-up campaign among 6–15 year olds. The red line represents the model-predicted overall effect of vaccination, while the green line represents the population direct effect of vaccination and the dotted blue line is the projected typhoid incidence in the absence of vaccination.

Following a one-time vaccine campaign among 6–15 year-old children, the incidence of typhoid in Vellore is predicted to decrease substantially within the first year and remain low for a period of 5–10 years (Figure 3*A*). The expected reduction in incidence depends on the vaccine efficacy (i.e. is greater for ViCV than for Ty21a), but exceeds that due to the direct protection alone for all three vaccines. However, the incidence of typhoid is projected to rebound to a level similar to or slightly above the average pre-vaccination incidence 7–17 years after vaccine introduction. The rebound occurs slightly sooner if vaccine-induced immunity wanes after 3 years (ViPS). However, the rebound in incidence is projected to occur even when there is no waning of clinical immunity, as we assume for Ty21a.

Routine vaccination of 6 year olds is predicted to lead to a less substantial reduction in typhoid incidence during the first year following introduction, but the decline is more sustained (Figure 3*B*). Typhoid incidence is predicted to decline by 26% over 20 years following introduction of Ty21a, and by 36–42% over 20 years following introduction of ViCV depending on the age at vaccination. ViPS is predicted to lead to the smallest long-term decline in incidence (11% decline over 20 years).

Routine vaccination along with a one-time catch-up campaign is predicted to lead to the largest decline in incidence (Figure 3*C*). However, when vaccine-induced immunity wanes, the incidence is again expected to rebound slightly after 10 or more years, particularly for ViPS, despite continued routine vaccination (Figure 3*C*).

**Figure 4:**
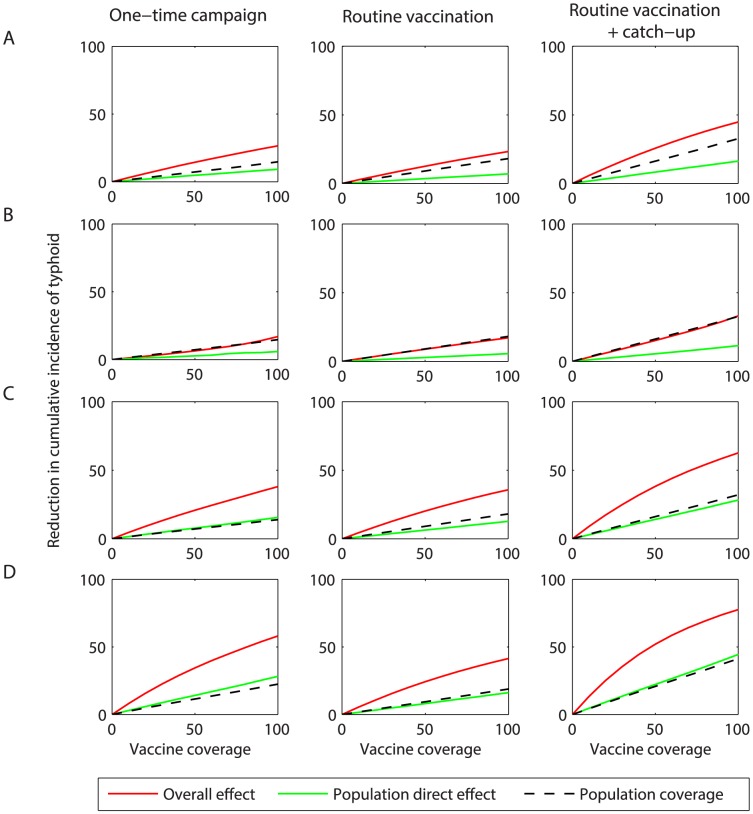
Overall effect of vaccination on the projected incidence of typhoid over the first 10 years following vaccine introduction. The model-predicted reduction in the cumulative incidence of typhoid at coverage levels ranging from 0 to 100% is plotted for vaccine-induced immunity and efficacy assumptions corresponding to (*A*) the Ty21a live oral vaccine, (*B*) the Vi-polysaccharide (ViPS) vaccine, and (*C*) the Vi-conjugate (ViCV) vaccine administered at 6 years of age or (*D*) 9 months of age. The red line represents the model-predicted overall effect of vaccination, while the green line represents the population direct effect of vaccination. The dotted black line represents the population coverage (i.e. the proportion of the population ever vaccinated).

The overall effectiveness over the first 10 years following vaccine introduction is expected to exceed the population direct effect for all coverage levels (Figure 4). Thus, vaccination is predicted to confer substantial indirect protection, particularly at coverage levels exceeding 40–50%. Administering ViCV at 9 months of age leads to an increase in the overall effectiveness for all scenarios, suggesting a considerable benefit could be derived from vaccinating infants.

The per-course impact of the different vaccination strategies is generally low (≤5 cases averted per 1,000 courses) during the first year following vaccine introduction, but increased with the duration of follow-up for most strategies (Table 2). Routine vaccination with ViCV at 9 months of age generally prevented the most cases per course compared to all other vaccination strategies.

**Table 2:** Cumulative number of typhoid cases prevented per 1,000 vaccine courses when vaccination is introduced with 80% coverage.

Vaccination scenario			Number of years post-introduction			
Vaccine	Age at routine vaccination	Age range of campaign	1 year	5 years	10 years	20 years
***One-time campaign***						
Ty21a		6–15 y	3.2	15.5	24.9	32.1
ViPS		6–15 y	4.6	14.2	13.0	8.9
ViCV		6–15 y	5.3	23.8	35.2	33.8
ViCV		9 m–15 y	4.6	21.4	34.5	35.7
***Routine vaccination***						
Ty21a	6 y		2.1	11.7	19.0	27.6
ViPS	6 y		3.7	12.1	13.8	11.2
ViPS	6, 9, 12 y		2.7	8.8	11.1	13.7
ViCV	6 y		4.2	19.9	29.8	38.1
ViCV	9 m		5.0	22.6	33.2	41.9
***Routine vaccination + catch-up campaign***						
Ty21a	6 y	6–15 y	3.0	12.9	20.1	28.2
ViPS	6 y	6–15 y	4.3	12.5	13.3	10.6
ViPS	6, 9, 12 y	6–15 y	3.7	9.2	11.4	14.1
ViCV	6 y	6–15 y	4.9	19.3	28.6	36.5
ViCV	9 m	9 m–15 y	4.4	17.9	27.8	37.0
ViCV	9 m, 6 y, 12 y	9 m–15 y	5.1	15.2	20.0	23.7

### Additional vaccination scenarios

**Figure 5:**
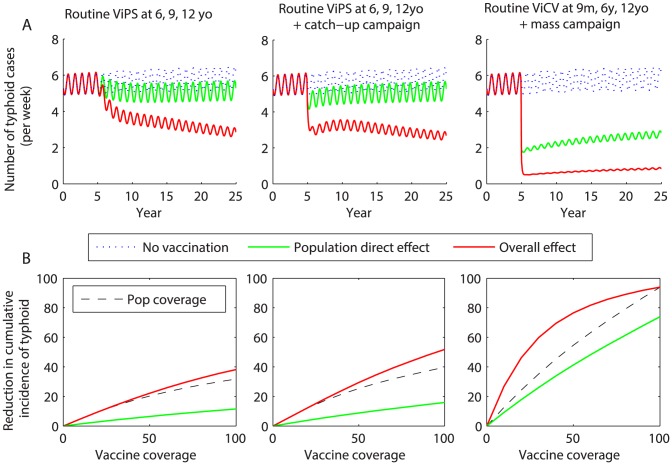
Impact and effectiveness of revaccination with Vi-based vaccines. Revaccination strategies include routine vaccination with ViPS at 6, 9, and 12 years of age; routine vaccination with ViPS of 6, 9, and 12 year olds plus a one-time catch-up campaign among 6–15 year olds; and routine vaccination with ViCV at 9 months, 6 years, and 12 years of age with a one-time mass vaccination campaign. (*A*) Impact of vaccination on the projected weekly incidence of typhoid. Vaccination is introduced in year 5 with 80% coverage. The red line represents the model-predicted overall effect of vaccination, while the green line represents the direct effect of vaccination and the dotted blue line is the projected typhoid incidence in the absence of vaccination. (*B*) The model-predicted reduction in the cumulative incidence of typhoid due to the overall (red line) and population direct (green line) effects of vaccination at coverage levels ranging from 0 to 100%, and the population coverage (black dotted line).

Revaccinating school-aged children every 3 years with ViPS is predicted to provide a substantial benefit compared to only vaccinating at 6 years of age, and prevents the incidence of typhoid from rebounding to pre-vaccination level (Figure 5). The overall effectiveness of vaccination is greater for all coverage levels when a catch-up campaign among school-aged children is also included, but the added benefit is lost 10–20 years following the campaign. While revaccinating with ViPS every three years initially prevented fewer cases per dose than only vaccinating once, the per-dose impact of revaccination was greater over 20 years of follow-up, since it prevented the incidence of typhoid from rebounding (Table 2).

An aggressive vaccination scenario consisting of a mass vaccination campaign using ViCV (targeting all age groups) followed by routine vaccination of 9 month, 6 year, and 12 year olds should lead to a >85% reduction in typhoid incidence at 80% coverage, but it is unlikely that typhoid would be eliminated (Figure 5). Mass vaccination with ViCV followed by routine vaccination of infants and school-aged children prevented fewer cases per dose compared to the other ViCV strategies (Table 2).

### Impact of improvements in sanitation

**Figure 6:**
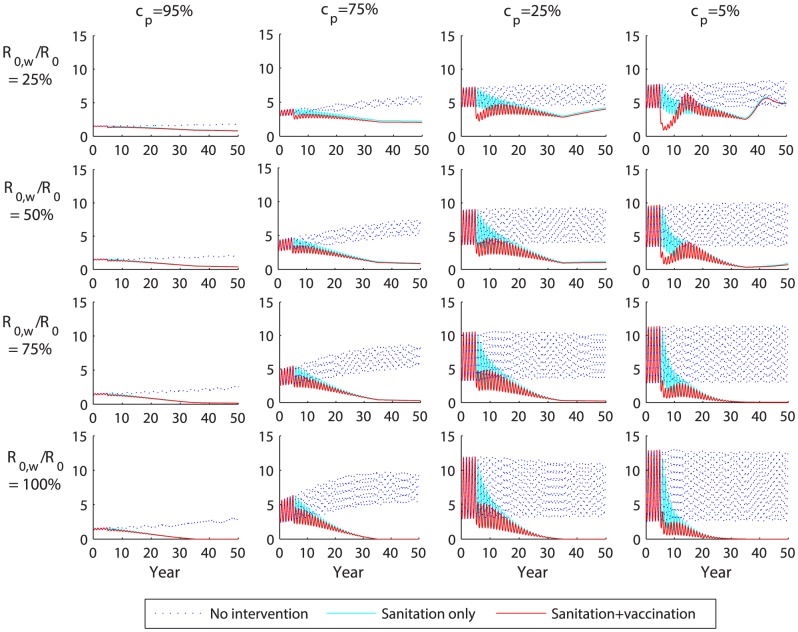
Combined impact of sanitation and vaccination on the projected weekly incidence of typhoid. The model-predicted weekly number of typhoid cases in Vellore is plotted for values of the proportion of transmission due to chronic carriers (*c_p_*) from 5% to 95% and the percent of transmission that is water-borne (*R*_0,*w*_/*R*_0_) from 25% to 100%. Improved sanitation is modeled as a reduction in water-borne transmission (*R*_0,*w*_) from baseline levels to zero over a 30-year period beginning in year 5. Vaccination is introduced in year 5 with 80% coverage as routine vaccination of 6 year olds plus a one-time catch-up campaign among 6–15 year olds using ViPS vaccines. The red line represents the overall effect of sanitation plus vaccination, while the light blue line represents the effect of improved sanitation only and the dotted blue line is the projected typhoid incidence in the absence of any intervention.

Combining vaccination with on-going improvements in sanitation is expected to lead to a sustained decline in typhoid incidence under most scenarios, and could potentially lead to elimination (Figure 6). When improvements in water quality/sanitation are modeled together with vaccination, most of the model-predicted decline in incidence comes from the impact of sanitation (Figure 6). Vaccination helps speed up the decline in incidence when carriers are less important (*c_p_*≤25%), and offers some additional benefit over sanitation alone in the long run when water-borne transmission is less important (*R*_0,*w*_/*R*_0_<50%). Under most assumptions about relative infectiousness of carriers and proportion of transmission that is water-borne, there is no appreciable rebound in incidence, with the exception of when *R*_0,*w*_/*R*_0_ = 25% and carrier are less important (*c_p_*≤25%). It may be possible to eliminate typhoid (i.e. decrease incidence to the point where stochastic fadeout would likely occur) through improvements in water quality/sanitation when *R*_0,*w*_/*R*_0_≥75%, or if *R*_0,*w*_/*R*_0_ = 50% and *c_p_ = *5%), (Figure 6).

## Discussion

The World Health Organization (WHO) currently recommends immunization of school-aged children against typhoid in endemic areas, but notes that the delivery strategy and vaccine type should depend on local considerations [6]. Country-level policymakers need to evaluate the relative merits of different typhoid vaccines and vaccination strategies. Mathematical modeling allows for a quantification of the expected long-term impact of different typhoid vaccination strategies, taking into account both the direct and indirect effects, and hence can be a helpful tool for policymakers.

All the typhoid vaccines we modeled were predicted to provide considerable short-term indirect protection. However, under certain scenarios, the incidence of typhoid is expected to rebound after a period of 5–15 years. This is in line with intuition given the build-up of susceptible individuals following a one-off campaign, since the indirect protection only serves to delay the time to infection of unvaccinated individuals. When vaccine-induced immunity wanes over time, it is possible that the incidence of typhoid during this rebound period could exceed the pre-vaccination incidence, although this has yet to be observed for typhoid. However, even successful routine vaccination with a fully immunizing vaccine (at a level below that necessary for elimination) can produce a “honeymoon period” after which cases rebound following the build-up of susceptibles, leading to a “post-honeymoon outbreak”, as has been observed for measles [26].

Following the introduction of school-based vaccination in Thailand in 1977, the incidence of typhoid declined by 43% in the first year and 94% after 10 years [7]. No rebound in incidence was observed, despite the discontinuation of the vaccination program in the early 1990s [8]. A number of factors may help explain why typhoid incidence has remained low. First, the duration of protection from the whole cell vaccine used in Thailand may be longer than for ViPS [27]. Second, the 7–12 year old population was targeted for vaccination each year, resulting in annual revaccination of school children [7]. Third, the vaccination campaign occurred following a widespread epidemic, which may have led to high levels of natural immunity in the population [7], [8]. Finally, on-going improvements in water quality, sanitation, and living conditions likely also played a role [8]. If we allow for a reduction in long-cycle transmission from current levels to zero over a 30-year period, our model predicts little to no rebound in incidence under most circumstances (Figure 6).

Our analysis suggests improvements in sanitation can have a more lasting impact than vaccination and eventually lead to the elimination of typhoid. However, in order to determine the specific benefit of improved water quality and sanitation in Vellore, we would need better estimates of the relative role of long-cycle (water-borne) versus short-cycle transmission and chronic carriers in this setting. While the best-fit model tended to emphasize short-cycle over long-cycle transmission in Vellore, the estimates of *R*_0,*w*_ and *R*_0,*p*_ were highly correlated (Figure S4). Furthermore, we did not account for improvements in water quality that may have been occurring in Vellore when fitting our model to the data. Epidemiological studies from Kathmandu, Nepal suggest that water-borne transmission may be the dominant modality in other settings [28]. A better understanding of how markers of improved water quality correlate with decreased transmission of typhoid is needed. History suggests that the introduction of filtration and chlorination of the water supply can lead to the eventual elimination of typhoid. An analysis of historical typhoid data from countries that successfully eliminated typhoid may help in this endeavor.

Previous attempts to model the impact of vaccination on the transmission dynamics of typhoid have been made, but these models did not take into account the characteristics of the current and next generation vaccines nor attempt to differentiate between the direct and indirect effects of vaccination [18]–[20]. Our results are qualitatively similar to those of Cvjetanovic *et al.* [18], yet their model considered mass vaccination using whole cell vaccines, which are no longer available. More broadly, this is to our knowledge the first parsimonious typhoid model explicitly fitted to an age-specific incidence time series from an endemic setting to examine the potential impact of vaccination.

Most if not all of the parameters we estimated by fitting our model to the data on typhoid patients from CMC hospital in Vellore are likely to be setting-specific. Therefore, our parameter estimates should not be over-interpreted or extrapolated to other settings. For example, we found that the age distribution of typhoid cases in Dhaka, Bangladesh was consistent with a higher basic reproductive number (*R*_0_∼7) in this setting [29]. Furthermore, we estimated the rate of waning immunity to clinical disease (*ε*) to be very low, consistent with another recent modeling study [21]. However, this may be due to the relatively high incidence of typhoid infection in this population leading to continual boosting of immunity through reinfections. Individuals have been known to experience more than one episode of clinical typhoid infection [30], so this finding should be interpreted cautiously. The development of clinical typhoid may depend on both the immune history of the individual as well as the infecting dose [31], but our parsimonious model does not explicitly account for the dose-response relationship. More extensive explorations of how model parameters vary by location and based on assumptions about model structure are warranted.

We found that assumptions about the relative infectiousness of chronic carriers, and hence the role they play in transmission, are critically important to the level of indirect protection expected from vaccination. Existing typhoid vaccines should have little impact on the carrier population, since chronic carriers already exhibit high levels of Vi antibodies [11]. Thus, vaccination only serves to decrease transmission from short-term clinically and subclinically infected individuals, and has only a small impact on carrier prevalence by preventing cases who could go on to become chronic carriers. If carriers are responsible for driving endemic typhoid transmission (*c_p_*>75%), then vaccination will afford little indirect protection for unvaccinated individuals.

The levels of indirect protection observed during trials conducted in Kolkata and other settings suggest that chronic carriers play a limited role in transmission [10], [16]. It is possible that differences in transmission from chronic carriers could explain the discrepant findings from Kolkata and Karachi [16], [17]. However, the low levels of overall and indirect protection observed in Karachi may have been biased by high rates of migration and diluted as a result of mixing between clusters [17].

We assume 0.3–10.1% of clinically and subclinically infected individuals become carriers depending on age at infection based on studies that suggest older individuals (women in particular) are more likely to become chronic typhoid carriers, possibly due to underlying gall bladder disease [32]–[35]. However, most of these studies are based on the follow-up of convalescent clinical typhoid cases who did not receive antibiotic treatment [33], [34]. It is possible that treated cases and subclinically infected individuals may be less likely to become chronic carriers. Furthermore, we assume that chronic carriage is life-long. Together, these may lead to overestimation of the prevalence of carriage predicted by our model (Figure S5, Text S1). If we assume a lower proportion of cases go on to become chronic carriers, the initial reduction in typhoid incidence following vaccine introduction was predicted to be bigger, but this was followed by a larger rebound in incidence (Figure S6). Thus, the overall effect of vaccination over longer time scales was similar (Figure S7). Similar results were obtained if we assumed the relative infectiousness of carriers is lower. There is a trade-off between the estimated relative infectiousness of carriers and the average duration of carriage, which is reflected in the equation for the proportion of transmission due to carriers (*c_p_*). Thus, our estimate for the prevalence and relative infectiousness of carriers (*r*) should not be over interpreted, but we believe the general conclusions are robust.

We advocate that elucidating the precise role of chronic carriers in transmission, and how it varies between settings, is a crucial research priority for understanding how to tackle local and regional elimination. Examining the genotypes of infecting typhoid bacteria may help shed light on the role of carriers in driving endemic transmission, since chronic carriers will tend to shed more ancestral strains [5]. Vaccine probe studies using a cluster-randomized design could also be used to correlate the role of chronic carriers with observed levels of indirect protection.

Country-level policymakers and international advisory boards will have to decide the best way to utilize existing typhoid vaccines and the soon-to-be-licensed ViCV. Mathematical modeling provides a powerful tool to test and compare different vaccination strategies, but the models must be rigorously fit to data and validated against early observations for the impact of vaccination. Our analysis suggests that both current and next-generation typhoid vaccines are likely provide substantial direct and indirect protection, but it is unlikely typhoid can be eliminated from a mid- to high-incidence setting such as Vellore through vaccination alone. Therefore, vaccination should be considered in conjunction with a suite of interventions, including improved treatment strategies, better detection and treatment of chronic carriers, and improvements in water and sanitation. We have provided a platform for understanding typhoid transmission dynamics that can be extended to other settings and used to assess additional control strategies.

## Supporting Information

Figure S1 **Diagram of typhoid model structure including vaccination.** (*A*) Model structure without vaccination (black lines) and vaccination with live-oral Ty21a (red lines), which is assumed to mimic natural immunity. (*B*) Vaccine-induced immunity for Vi-based vaccines (ViPS and ViCV) is assumed to be distinct from natural immunity and “all-or-nothing”. The compartments, arrows, and rates describing vaccination are in red. All other rates are as described in (*A*).
(TIF)

Figure S2 **Comparison between model-predicted vaccine efficacy and observed vaccine efficacy for Vi-polysaccharide vaccines and Vi-conjugate vaccines.** The red line represents the model-predicted direct effect of vaccination when coverage is low (10%) during year 1–5 of follow-up, while the black circles and lines represent the summarized observed vaccine efficacy and 95% confidence interval [9] for (*A*) Vi-polysaccharide (ViPS) vaccines and (*B*) Vi-conjugate (ViCV) vaccines.
(EPS)

Figure S3 **Weekly incidence and age distribution of typhoid and paratyphoid inpatients at Christian Medical College hospital in Vellore, India.** (*A*) Number of patients discharged with a diagnosis of typhoid (blue) or paratyphoid (green) per week between January 2000 and February 2012. (*B*) Average number of typhoid and paratyphoid cases per week of the year (blue and green lines, respectively) along with average monthly rainfall in Vellore (black line, in millimetres). (*C*) Proportion of typhoid and paratyphoid cases by age (in 5-year age groups).
(EPS)

Figure S4 **Likelihood profiles for estimated model parameters.** The negative log-likelihood is plotted in blue for parameter values around the estimated value for the best-fit model (blue asterisk) and within the constrained parameter ranges. The black dotted line represents the cut-off value for the 95% confidence intervals, corresponding to a chi-squared distribution with 1 degree of freedom. The negative log-likelihood when varying *R*_0,*p*_ and *R*_0,*w*_ together (holding *qR*_0,*w*_ constant) is also plotted.
(EPS)

Figure S5 **Prevalence of chronic carriers by age predicted by the model.** Mean proportion of the population in age class *a* predicted to be in the *C* state for (*A*) the baseline model fitted to the typhoid data from Vellore, India, and (*B*) the model assuming the proportion of infections leading to chronic carriage was reduced by 90% (i.e. *θ* = 0.0003–0.0101 depending on age).
(EPS)

Figure S6 **Predicted impact of vaccination assuming the probability of becoming a chronic carrier following infection is reduced by 90%.** Vaccination is introduced in year 5 with 80% coverage as (*A*) a one-time campaign among 6–15 year olds, (*B*) routine vaccination of 6 year olds, or (*C*) routine vaccination of 6 year olds plus a one-time catch-up campaign among 6–15 year olds. The red line represents the model-predicted overall effect of vaccination, while the green line represents the population direct effect of vaccination and the dotted blue line is the projected typhoid incidence in the absence of vaccination.
(EPS)

Figure S7 **Overall effect of vaccination on the projected incidence of typhoid assuming the probability of becoming a chronic carrier following infection is reduced by 90%.** The model-predicted reduction in the cumulative incidence of typhoid over the first 10 years following vaccine introduction at coverage levels ranging from 0 to 100% is plotted for vaccine-induced immunity and efficacy assumptions corresponding to (*A*) the Ty21a live oral vaccine, (*B*) the Vi-polysaccharide (ViPS) vaccine, and (*C*) the Vi-conjugate (ViCV) vaccine administered at 6 years of age or (*D*) 9 months of age. The red line represents the model-predicted overall effect of vaccination, while the green line represents the population direct effect of vaccination. The dotted black line represents the population coverage (i.e. the proportion of the population ever vaccinated).
(EPS)

Figure S8 **Overall effectiveness of different typhoid vaccination strategies in a high incidence setting.** (*A*) Age distribution of typhoid cases in Dhaka, Bangladesh [29] and predicted by the model with *R*_0_ = 7. We assume the reporting rate is 50% lower in infants <1 year of age. (*B*) Percent reduction in the cumulative incidence of typhoid due to the overall effect of vaccination at 80% coverage. The bars show the model-predicted reduction in typhoid incidence over the first 1 year, 5 years, 10 years, and 20 years following vaccine introduction. The white bars represent school-based vaccination assumptions corresponding to the Ty21a live oral vaccine; the light grey bars represent the Vi-polysaccharide (ViPS) vaccine; the medium grey bars represent revaccination every three years with ViPS; the dark grey bars represent Vi-conjugate (ViCV) vaccine administered at 6 years of age; and the black bars represent the ViCV vaccine administered at 9 months of age.
(EPS)

Table S1 **Sensitivity of estimated parameters to fixed parameter assumptions.**
(PDF)

Table S2 **Summary of vaccination parameters.**
(PDF)

Text S1 **Detailed methods.**
(PDF)
