# Supplementary figures and images for: Predicting the Impact of Vaccination on the Transmission Dynamics of Typhoid in South Asia: A Mathematical Modeling Study
Source: PLoS Negl Trop Dis. 2014 Jan 9;8(1):e2642. doi: 10.1371/journal.pntd.0002642 (PMC3886927; doi:10.1371/journal.pntd.0002642)

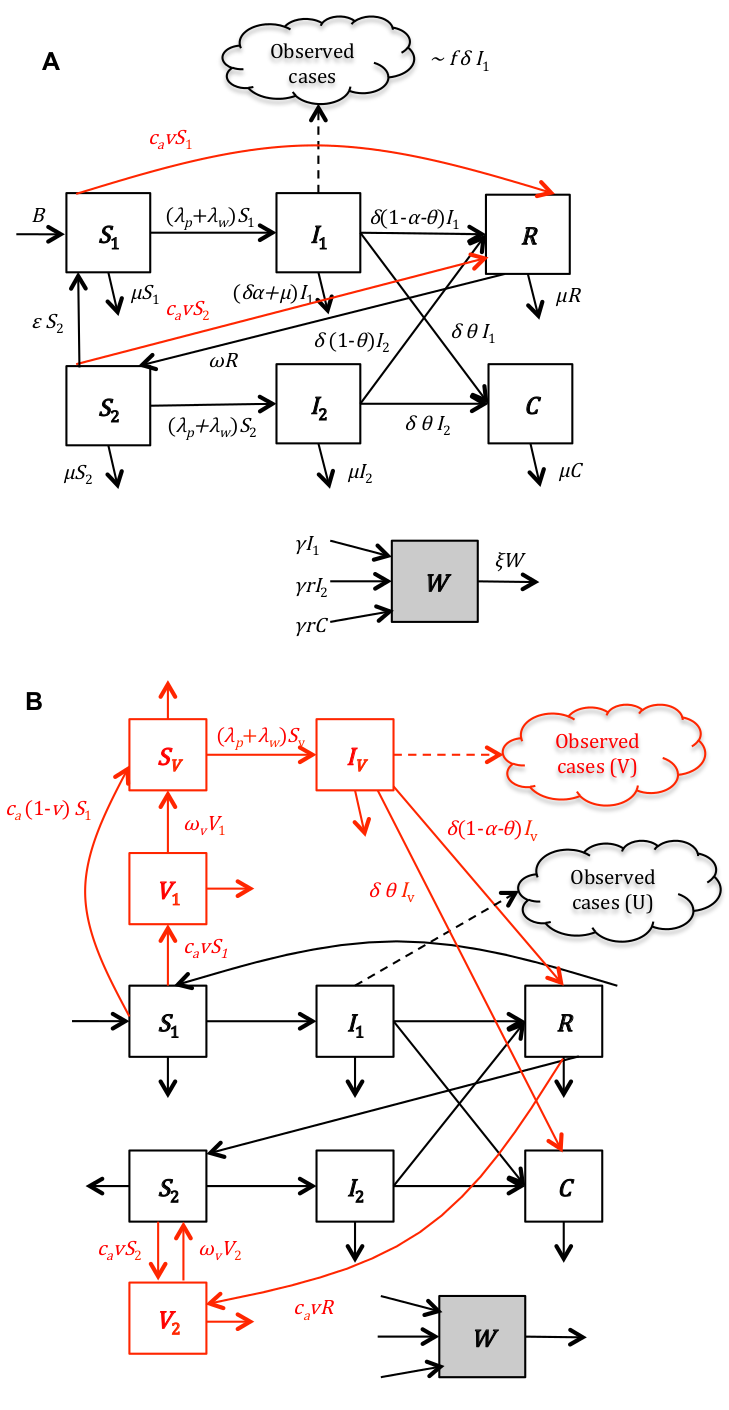

Supplement: Figure S1 — Diagram of typhoid model structure including vaccination. (A) Model structure without vaccination (black lines) and vaccination with live-oral Ty21a (red lines), which is assumed to mimic natural immunity. (B) Vaccine-induced immunity for Vi-based vaccines (ViPS and ViCV) is assumed to be distinct from natural immunity and “all-or-nothing”. The compartments, arrows, and rates describing vaccination are in red. All other rates are as described in (A). (TIF) [file pntd.0002642.s001.tif]
