# Supplementary material for: Predicting the Impact of Vaccination on the Transmission Dynamics of Typhoid in South Asia: A Mathematical Modeling Study
Source: PLoS Negl Trop Dis. 2014 Jan 9;8(1):e2642. doi: 10.1371/journal.pntd.0002642 (PMC3886927; doi:10.1371/journal.pntd.0002642)
Supplement: Table S1 — Sensitivity of estimated parameters to fixed parameter assumptions. (PDF) [file pntd.0002642.s009.pdf]

Table S1. Sensitivity of estimated parameters to fixed parameter assumptions. The log-likelihood of the best-fit model with the original parameter assumptions is -6,379.

**Fixed parameter**	**Original value**	**New value**	**Estimated parameters**	**Log-likelihood**
Duration of full immunity from infection (1/ω)	104 weeks	4 weeks	$R_{0,p}$ = 2.46<br>$R_{0,w}$ = 0.23<br>q = 0.82<br>ϕ = 18.8 weeks<br>ε = 2.86x10^{-7}<br>r = 0.01<br>f = 0.0053	-6,380
Duration of full immunity from infection (1/ω)	104 weeks	50 years	$R_{0,p}$ = 1.82<br>$R_{0,w}$ = 1.26<br>q = 0.19<br>ϕ = 16.8 weeks<br>ε = 2.49x10^{-6}<br>r = 0.01<br>f = 0.0052	-6,377
Duration of infectiousness (1/δ)	4 weeks	2 weeks	$R_{0,p}$ = 2.80<br>$R_{0,w}$ = 0.17<br>q = 0.96<br>ϕ = 22.5 weeks<br>ε = 4.71x10^{-13}<br>r = 0.01<br>f = 0.0050	-6,388
Duration of infectiousness (1/δ)	4 weeks	8 weeks	$R_{0,p}$ = 2.67<br>$R_{0,w}$ = 4.57x10^{-5}<br>q = 0.79<br>ϕ = 45.9 weeks<br>ε = 1.61x10^{-8}<br>r = 0.01<br>f = 0.0055	-6,389
Fraction infected who become carriers (θ)	0.003-0.101 depending on age	0.0003-0.0101 depending on age	$R_{0,p}$ = 2.23<br>$R_{0,w}$ = 0.24<br>q = 0.66<br>ϕ = 15.2 weeks<br>ε = 5.40x10^{-9}	-6,366

			$r = 0.01$<br>$f = 0.0058$	
Fraction infected who become carriers (θ)	0.003-0.101 depending on age	0.006-0.202 depending on age	$R_{0,p} = 2.85$<br>$R_{0,w} = 0.23$<br>$q = 1.00$<br>$\phi = 19.8$ weeks<br>$\varepsilon = 3.14 \times 10^{-11}$<br>$r = 0.01$<br>$f = 0.0050$	-6,387
Rate of decay of infectious particles from water supply (ξ)	1/3 week^{-1}	1 week^{-1}	$R_{0,p} = 2.29$<br>$R_{0,w} = 0.48$<br>$q = 0.004$<br>$\phi = 52.5$ weeks<br>$\varepsilon = 7.45 \times 10^{-9}$<br>$r = 0.01$<br>$f = 0.0051$	-6,395
Rate of decay of infectious particles from water supply (ξ)	1/3 week^{-1}	1/9 week^{-1}	$R_{0,p} = 2.77$<br>$R_{0,w} = 0.001$<br>$q = 0.014$<br>$\phi = 9.9$ weeks<br>$\varepsilon = 9.69 \times 10^{-9}$<br>$r = 0.01$<br>$f = 0.0053$	-6,394
