# Supplementary material for: Predicting the Impact of Vaccination on the Transmission Dynamics of Typhoid in South Asia: A Mathematical Modeling Study
Source: PLoS Negl Trop Dis. 2014 Jan 9;8(1):e2642. doi: 10.1371/journal.pntd.0002642 (PMC3886927; doi:10.1371/journal.pntd.0002642)
Supplement: Table S2 — Summary of vaccination parameters. (PDF) [file pntd.0002642.s010.pdf]

Table S2. Summary of vaccination parameters.

**Parameter**	**Symbol**	**Value**	**Source***
Ty21a			
Vaccine efficacy at time of inoculation	v	48%	[6]
Duration of vaccine-induced immunity	$1/\omega_v$	Same as natural immunity	[5]
ViPS			
Vaccine efficacy at time of inoculation	v	80%	[6]
Duration of vaccine-induced immunity	$1/\omega_v$	3 years	[6]
ViCV			
Vaccine efficacy at time of inoculation	v	95.6%	[6,8]
Duration of vaccine-induced immunity	$1/\omega_v$	19.2 years	[9]

*Sources correspond to references in Text S1.
