# Supplementary material for: Predicting the Impact of Vaccination on the Transmission Dynamics of Typhoid in South Asia: A Mathematical Modeling Study
Source: PLoS Negl Trop Dis. 2014 Jan 9;8(1):e2642. doi: 10.1371/journal.pntd.0002642 (PMC3886927; doi:10.1371/journal.pntd.0002642)
Supplement: Text S1 — Detailed methods. (PDF) [file pntd.0002642.s011.pdf]

SUPPORTING INFORMATION

Predicting the Impact of Vaccination on the Transmission Dynamics of Typhoid in South Asia: A Mathematical Modeling Study

Virginia E. Pitzer, ScD[1,2], Cayley C. Bowles, AB[3,4], Stephen Baker, PhD[5,6,7], Gagandeep Kang, MD[8], Veeraraghavan Balaji, MD[8], Jeremy J. Farrar, MD[4,5,6], Bryan T. Grenfell, PhD[2,4]

[1]*Department of Epidemiology of Microbial Diseases, Yale School of Public Health, New Haven, CT USA*

[2]*Fogarty International Center, National Institutes of Health, Bethesda, MD USA*

[3]*Department of Epidemiology, Harvard School of Public Health, Boston, MA USA*

[4]*Department of Ecology and Evolutionary Biology, Princeton University, Princeton, NJ USA*

[5]*The Hospital for Tropical Diseases, Wellcome Trust Major Overseas Programme, Oxford University Clinical Research Unit, Ho Chi Minh City, Vietnam*

[6]*Centre for Tropical Medicine, Nuffield Department of Clinical Medicine, Oxford University, United Kingdom*

[7]*The London School of Hygiene and Tropical Medicine, London, United Kingdom*

[8]*Department of Gastrointestinal Sciences, Christian Medical College, Vellore, Tamil Nadu, India*

Text S1 Contents

1. Model description and equations
2. Derivation of the basic reproductive number (R_0)
3. Model fitting procedure
4. Estimation of demographic parameters for Vellore district
5. Modeling vaccination
6. Calculation of direct, indirect, and total effects
7. Impact of vaccination in other settings

1. Model description and equations

Our model assumes individuals are born (at a rate B) susceptible to clinical typhoid infection (S_1), which occurs at a rate ($\lambda_p + \lambda_w$), where λ_p and λ_w are the prevalent-case and water-borne forces of infection, respectively. Individuals with "primary infection" (I_1) contribute to both "short-cycle" (prevalent-case) transmission via contamination of food, drinking water, etc in the immediate environment and "long-cycle" (water-borne) transmission via contamination of the water supply (W). Infectious individuals are assumed to shed bacteria into the water supply at a rate γ, and infectious bacteria lose viability (i.e. are removed from the water supply) at a rate ξ. Seasonality in transmission was assumed to act upon long-cycle but not short-cycle transmission.

Infectious individuals recover at a rate δ. We assume most recovered individuals are temporarily immune to reinfection (R), while a small fraction (θ) goes on to become chronic (life-long) asymptomatic carriers of typhoid (C), or experience disease-induced mortality (α). We assume individuals lose their immunity at a rate ω and become susceptible to future subclinical infection (S_2) (i.e. short-term carriage), but are immune to clinical reinfection. This immunity to clinical reinfection can wane at a rate ε, and individuals can reenter the fully susceptible state (S_1). We differentiate between susceptibility to clinical and subclinical infection because the age distribution of typhoid cases in Vellore is suggestive of fairly strong immunity to clinical infection in this setting (Figure S3), but reinfections with typhoid are known to occur and likely contribute to transmission [1,2]. Thus, the model allows for boosting of immunity through repeated subclinical infections. We assume subclinical infectious individuals (I_2) can also go on to become chronic carriers. Both short-term and long-term carriers are assumed to contribute to transmission at a relative rate r compared to primary infections.

The model equations are as follows:

$$\frac{dS_1}{dt} = B + \varepsilon S_2 - (\lambda_p + \lambda_w)S_1 - \mu S_1$$

$$\frac{dI_1}{dt} = (\lambda_p + \lambda_w)S_1 - \delta I_1 - \mu I_1$$

$$\frac{dR}{dt} = \delta(1 - \theta - \alpha)(I_1 + I_2) - \omega R - \mu R$$

$$\frac{dC}{dt} = \delta\theta(I_1 + I_2) - \mu C$$

$$\frac{dS_2}{dt} = \omega R - \varepsilon S_2 - (\lambda_p + \lambda_w)S_2 - \mu S_2$$

$$\frac{dI_2}{dt} = (\lambda_p + \lambda_w)S_2 - \delta I_2 - \mu I_2$$

$$\frac{dW}{dt} = \gamma(I_1 + rI_2 + rC) - \xi W$$

where B are new births, μ is the natural mortality rate, ω is the rate of waning natural immunity to infection, ε is the rate of waning clinical immunity (i.e. rate of returning to full susceptibility to clinical disease), δ is the rate of recovery from infectiousness, θ is the fraction of infectious individuals who go on to become carriers (which varies by age), α is the disease-induced mortality rate, γ is the rate of shedding into the water supply, and ξ is the rate of decay of infectious particles from the water supply. The short-cycle (person-to-person) and long-cycle (water-borne) forces of infection are given by:

$$\lambda_p = \frac{\beta_p(I_1 + rI_2 + rC)}{N},$$

$$\lambda_w = \beta_w(1 + q\cos(2\pi(t - \phi)))W,$$

where β_p and β_w are the short-cycle and long-cycle transmission rates, respectively, r is the relative infectiousness of subclinical infections (I_2) and chronic carriers (C), N is the population size ($= S_1 + I_1 + R + C + S_2 + I_2$), q is the amplitude of seasonal forcing, and ϕ is the seasonal offset parameter. Short-cycle transmission was assumed to be frequency-dependent, while long-cycle transmission was assumed to be density dependent [3].

We incorporated age structure into the model to compare model output to age-stratified incidence data and to incorporate age-specific vaccination strategies. Thus, each epidemiological model compartment (with the exception of W) is actually composed of a number of age-specific compartments, e.g. $\boldsymbol{S_1} = \{S_{1,1}, S_{1,2},\ldots, S_{1,a}\}$. Transmission-relevant mixing was assumed to be homogeneous with respect to age. Natural mortality was assumed to occur from all epidemiological states and age groups at a rate μ, leading to a pyramidal age structure that is representative of Vellore.

2. Derivation of the basic reproductive number (R_0)

The basic reproductive number (R_0), defined as the expected number of secondary infections produced by an infectious individual in a fully susceptible population, for our model is:

$$R_0 = \frac{1}{\mu + \delta}\left(\beta_p + \frac{\gamma\beta_w}{\xi}\right)\left(1 + \frac{\delta\theta r}{\mu}\right),$$

which is the product of the duration of infectiousness ($1/(\delta+\mu)$) and the rate of short-cycle (β_p) and long-cycle transmission ($\gamma\beta_w/\xi$) for both primary infections and the fraction θ who become chronic carriers (weighted mean of θ_a across the age distribution of the population), scaled by their relative transmissibility, r, and duration of infectiousness, δ/μ.

We used the next generation matrix method of van den Driessche and Watmough [4] to derive the expression for the basic reproductive number (R_0) as a function of the model parameters. We define the rate of change in the infectious compartments at the disease-free equilibrium to be:

$$\dot{\mathbf{X}}_S = \left. \begin{pmatrix} \dot{I}_1 \\ \dot{I}_2 \\ \dot{C} \\ \dot{W} \end{pmatrix} \right|_{X_0} = \mathscr{F}(X) - \mathscr{V}(X)$$

where $X_0 = \{ I_1=0, I_2=0, C=0, W=0, S_1=N, S_2=0, R=0\}$. The basic reproductive number (R_0) is equal to the maximum eigenvalue of the next generation matrix, FV^{-1}, where

$$F = \frac{\partial \mathscr{F}_i}{\partial X_j} = \begin{pmatrix} \beta_p & r\beta_p & r\beta_p & \beta_w N \\ 0 & 0 & 0 & 0 \\ 0 & 0 & 0 & 0 \\ 0 & 0 & 0 & 0 \end{pmatrix}$$

and

$$V = \frac{\partial \mathscr{V}_i}{\partial X_j} = \begin{pmatrix} \delta+\mu & 0 & 0 & 0 \\ 0 & \delta+\mu & 0 & 0 \\ -\delta\theta & -\delta\theta & \delta+\mu & 0 \\ -\gamma & -r\gamma & -r\gamma & \xi \end{pmatrix}$$

Therefore,

$$FV^{-1} = \begin{pmatrix} R_0 & * & * & * \\ 0 & 0 & 0 & 0 \\ 0 & 0 & 0 & 0 \\ 0 & 0 & 0 & 0 \end{pmatrix}$$

where

$$R_0 = \frac{1}{\mu+\delta}\left(\beta_p + \frac{\gamma\beta_w}{\xi}\right)\left(1+\frac{\delta\theta r}{\mu}\right).$$

This expression can be broken down to show the independent contributions of short-cycle and long-cycle transmission. It is possible to rewrite $R_0 = R_{0,p} + R_{0,w}$, where the basic reproductive numbers of short-cycle ($R_{0,p}$) and long-cycle ($R_{0,w}$) transmission are given by

$$R_{0,p} = \frac{\beta_p}{\mu+\delta}\left(1+\frac{\delta\theta r}{\mu}\right)$$

and

$$R_{0,w} = \frac{\gamma\beta_w}{(\mu+\delta)\xi}\left(1+\frac{\delta\theta r}{\mu}\right).$$

The proportion of transmission from carriers versus symptomatic infections (c_p) can be calculated as

$$c_p = \frac{\delta\theta r}{\mu+\delta\theta r}.$$

3. Model fitting procedure

Observed typhoid hospitalizations in age group a at week w ($x_{a,w}$) were assumed to represent a fraction of the model-predicted number of primary infections at time $t = w$ ($\hat{x}_{a,w} = fI_{1,a}(t = w)$), where f is the reporting fraction. The reporting fraction f takes into account the probability that an individual with clinical typhoid infection in Vellore will seek care at (or be referred to) Christian Medical College (CMC) hospital, be admitted as an in-patient, and be culture-confirmed; hence, it takes into account many factors, including the probability of primary infection leading to clinical typhoid (which we assume is roughly 10%) [1], treatment-seeking behavior, and culture sensitivity

(which is relatively poor). We assumed the observed number of reported typhoid cases in age group a was Poisson-distributed with a mean equal to the model-predicted number of cases occurring at that time.

The log-likelihood ($\log(L)$) of the model was given by the equation:

$$\log(L) = \sum_{w=w_0}^{w_f} \sum_{a=1}^{a_{max}} \left(-\hat{x}_{a,w} + x_{a,w} \log \hat{x}_{a,w} - \sum_{j=1}^{x_{a,w}} \log j \right)$$

We fit the model by minimizing the negative log-likelihood after an initial burn-in period of 50 years, which was long enough to reach the epidemiological quasi-steady state (i.e. ignoring seasonality). We used the "fminsearch" command in MATLAB v7.14 (MathWorks, Natick, MA) to minimize the $-\log(L)$, which employs a direct simplex search method.

The best-fitting parameter vector was found to be very sensitive to the starting conditions (i.e. initial parameter "guess"). Therefore, we used Latin Hypercube Sampling (LHS) to sample evenly from the range of plausible parameter distributions. We first examined the $-\log(L)$ over the full range of each parameter (or pair of parameters in the case of $R_{0,p}$ and $R_{0,w}$), using LHS to sample the remaining parameters. This allowed us to further localize some of the initial parameter distributions. We then drew 1,000 LHS samples from the localized parameter distributions, calculated the $-\log(L)$, and ranked the LHS parameter samples according to the lowest values. We chose the 10 best parameter vectors, and used these to initialize the model-fitting process. We iterated the search process for the minimum $-\log(L)$ twice, using the output from the first search to initiate the second search in order to decrease the probability of obtaining a local (versus global) minimum value. The parameter set corresponding to the lowest $-\log(L)$ value was then selected as the best-fitting model.

The likelihood profile around each of the best-fit parameters was calculated while holding the other parameters fixed. We compared the likelihood profile to a chi-square

distribution with one degree of freedom to construct 95% confidence intervals for each parameter. The likelihood profile when varying $R_{0,p}$ and $R_{0,w}$ together (while holding $qR_{0,w}$ constant) was also calculated.

We analyzed the sensitivity of the estimated parameters to the fixed parameter assumptions by varying the fixed parameters one at a time to a plausible high and low value, then refitting the model (Table S1).

Despite the relative simplicity of our model and the limited number of parameters that we attempted to estimate, some of the estimated parameters are not well identified (i.e. the likelihood profiles are relatively flat) (Figure S4). Epidemiological studies are needed to better identify some of the unknown parameters, including the relative infectiousness of carriers (r), the contribution of short- and long-cycle transmission ($R_{0,p}$ and $R_{0,w}$), and how such transmission varies seasonally (q and ϕ).

4. Estimation of demographic parameters for Vellore district

We estimated weekly number of births from July 1971 to February 2012 based on the crude birth rate for Tamil Nadu state and the total number of live births and deaths in Vellore district for 1997 to 2002 (http://www.indiastat.com). The district population size for 1997-2002 was back-calculated from both the birth and death rates and compared to the actual district population size from the 2001 census. The district population size for the remaining years was calculated by assuming $N_{y+1} = N_y\,(1+b\text{-}d)$, where N_y is the district population size in year y, b is the crude annual birth rate, and d is the crude annual death rate. Finally, the number of weekly births (B) was interpolated assuming the number of births on 1 July of each year was equal to the crude birth rate times the estimated population size for that year divided by 52.2 weeks.

We compared the simulated population size and age structure to data for Vellore district to verify that the model was able to accurately reproduce the population demographics.

Since CMC hospital is a referral facility, a large proportion of patients may be referred from other hospitals, possibly in other states. Such patients are typically not suffering from acute illness. These non-acute referral patients may bias the patterns of the seasonal occurrence of incident typhoid infections in Vellore. However, the length of the time series and detailed data available from Vellore is unique and essential to estimating key model parameters, and the overall patterns are still likely to be representative of typhoid transmission in Vellore and the surrounding regions.

5. Modeling vaccination

To model the expected impact of vaccination using live oral vaccine (Ty21a), we assume vaccine-induced immunity mimics natural immunity (Figure S1*a*). A fraction $c_a v$ of vaccinated susceptible individuals (in S_1 or S_2) is moved to the immune (R) class, where c_a is the coverage level for vaccination at age a and v is the vaccine efficacy. Vaccinated individuals are assumed to be temporarily protected from typhoid infection, and then lose this full immunity at a rate ω; most individuals become susceptible to subclinical infection only (S_2), but a small fraction (ε) may return to full susceptibility to clinical infection (S_1). Since the estimate of ε was not significantly different from zero, we fixed $\varepsilon = 0$ when analyzing the impact of vaccination to aid in the interpretability of the model (i.e. we assumed no waning of clinical immunity) [5]. We assumed the vaccine efficacy for Ty21a was 48% in accordance with the cumulative efficacy over 2.5 to 3 years in a recent meta-analysis [6] (Table S2).

For the Vi-based vaccines, we model vaccine-induced immunity as distinct from natural immunity (Figure S1*b*). In this case, we assume a fraction $c_a v$ of susceptible and recovered individuals (in S_1, S_2, or R) is moved to a corresponding vaccinated class (V_1 or V_2). We assume vaccine protection is "all-or-nothing"; results assuming "leaky" protection were similar [7]. Vaccinated individuals lose their immunity and wane back to the corresponding susceptible state at a rate ω_v. For ViPS, we assumed an initial vaccine

efficacy of 80% and a mean duration of protection of 3 years, while for ViCV, we assumed an initial efficacy of 95.6% and a duration of 19.2 years, based on a comparison between the predicted direct effect and the waning of vaccine efficacy observed during trials [6,8,9] (Figure S2, Table S2).

We implemented the vaccination campaigns over a period of four weeks by assuming that a fraction $1\text{-}(1\text{-}c_a)^{1/4}$ of susceptible individuals were effectively immunized each week. Routine vaccination was implemented as part of the aging process, e.g. was assumed to occur upon movement into the 6-year age group.

6. Calculation of direct, indirect, and total effects

To model the Vi-based vaccines, separate S_V and I_V compartments were enumerated to keep track of vaccinated individuals who were previously susceptible to clinical infection in order to calculate the direct, indirect, and total effects of vaccination [10], but these compartments are equivalent to the S_1 and I_1 compartments in terms of the dynamics. The direct (DE_y), indirect (IE_y), and total effect (TE_y) of vaccination in year y of follow-up were calculated as follows:

$$DE_y = 1 - \frac{\sum_{t=t_v+52(y-1)}^{t_v+52y} \sum_a z_{a,t} / \widetilde{V_{a,t}}}{\sum_{t=t_v+52(y-1)}^{t_v+52y} \sum_a u_{a,t} / \left(N_{a,t} - \widetilde{V_{a,t}}\right)},$$

$$IE_y = 1 - \frac{\sum_{t=t_v+52(y-1)}^{t_v+52y} \sum_a u_{a,t} / \left(N_{a,t} - \widetilde{V_{a,t}}\right)}{\sum_{t=t_v+52(y-1)}^{t_v+52y} \sum_a x_{a,t} / N_{a,t}},$$

$$TE_y = 1 - \frac{\sum_{t=t_v+y-1}^{t_v+y} \sum_a z_{a,t} / \widetilde{V_{a,t}}}{\sum_{t=t_v+52(y-1)}^{t_v+52y} \sum_a x_{a,t} / N_{a,t}},$$

where $z_{a,t}$ and $u_{a,t}$ are the number of typhoid cases of age a at time t among vaccinated versus unvaccinated individuals, respectively, $x_{a,t}$ is the model-predicted incidence in the absence of vaccination, $\widetilde{V_{a,t}}$ is the number of individuals of age a at time t who have ever been vaccinated, $N_{a,t}$ is the total number of individuals of age a in the population at time t, and t_v is the time of vaccine introduction (in weeks).

Population direct effect of vaccination

We define the population direct effect of vaccination as the expected reduction in typhoid incidence in the population if vaccination provides only direct protection for vaccinated individuals. The population direct effect t weeks after vaccine introduction ($PopDE_t$) is calculated using the equation:

$$PopDE_t = \frac{\sum_a V_{a,t} x_{a,t} * v}{\sum_a x_{a,t}}$$

where $V_{a,t}$ ($=V_1+V_2$) is the expected number of individuals of age a with vaccine-derived immunity at time t (which takes into account current and past vaccination coverage and waning of vaccine-induced immunity), $x_{a,t}$ is the expected typhoid incidence in age group a at week t in the absence of vaccination ($=f\,I_{1,a,t}$), and v is the vaccine efficacy.

7. Impact of vaccination in other settings

The expected impact of the various vaccination strategies we examined will likely depend upon the underlying age-incidence pattern of clinical typhoid cases. For instance, the age distribution of clinical typhoid cases was found to be considerably younger in Dhaka, Bangladesh compared to Vellore [11]. This is likely reflective of a higher typhoid transmission rate. It is possible to reproduce the age distribution of typhoid cases in Dhaka by increasing the R_0 in our model to ~7 (Figure S8).

As a result of the higher transmission rate, the overall effectiveness of typhoid vaccination predicted by our model is lower in Dhaka compared to Vellore, particularly for vaccine strategies targeting school-aged children. Furthermore, the benefit of a ViCV vaccine capable of providing protection for infants is greater in Dhaka (Figure S8). These are some of the factors that need to be considered when determining the best vaccination strategy for a given location.

References

1. Hornick R, Greisman S, Woodward TE, Dupont HL, Dawkins AT, Snyder MJ (1970) Typhoid fever: Pathogenesis and immunologic control (Part 1). N Engl J Med 283: 686–691.

2. Hornick R, Greisman S, Woodward TE, Dupont HL, Dawkins AT, Snyder MJ (1970) Typhoid fever: Pathogenesis and immunologic control (Part 2). N Engl J Med 283: 739–746.

3. McCallum H, Barlow N, Hone J (2001) How should pathogen transmission be modelled? Trends Ecol Evol 16: 295–300.

4. Van den Driessche P, Watmough J (2002) Reproduction numbers and sub-threshold endemic equilibria for compartmental models of disease transmission. Math Biosci 180: 29–48.

5. Levine MM, Ferreccio C, Abrego P, Martin OS, Ortiz E, Cryz S (1999) Duration of efficacy of Ty21a, attenuated Salmonella typhi live oral vaccine. Vaccine 17 Suppl 2: S22–7.

6. Fraser A, Goldberg E, Acosta C (2009) Vaccines for preventing typhoid fever (Review). The Cochrane Library. Chichester: John Wiley & Sons.

7. Halloran ME, Haber M, Longini IM (1992) Interpretation and estimation of vaccine efficacy under heterogeneity. Am J Epidemiol 136: 328–343.

8. Lin F, Ho V, Khiem H (2001) The efficacy of a Salmonella Typhi Vi conjugate vaccine in two-to-five-year-old children. N Engl J Med 344: 1263–1269.

9. Lanh MN, Bay P Van, Ho VA, Tran CT, Lin FYC, Bryla D a, Chu C, Schiloach J, Robbins JB, Schneerson R, Szu SC (2003) Persistent efficacy of Vi conjugate vaccine against typhoid fever in young children. N Engl J Med 349: 1390–1391. doi:10.1056/NEJM200310023491423.

10. Halloran ME, Longini IM, Struchiner CJ (1999) Design and interpretation of vaccine field studies. Epidemiol Rev 21: 73–88.

11. Saha SK, Baqui AH, Hanif M, Darmstadt GL, Ruhulamin M, Nagatake T, Santosham M, Black RE (2001) Typhoid fever in Bangladesh: Implications for vaccination policy. Pediatr Infect Dis J 20: 521–524.
